# Emerging Applications of Drug Delivery Systems in Oral Infectious Diseases Prevention and Treatment

**DOI:** 10.3390/molecules25030516

**Published:** 2020-01-24

**Authors:** Jingou Liang, Xinyu Peng, Xuedong Zhou, Jing Zou, Lei Cheng

**Affiliations:** State Key Laboratory of Oral Diseases& West China School of Stomatology& National Clinical Research Center for Oral Diseases, Sichuan University, Chengdu 610041, China; liangjingoudentist@163.com (J.L.); pengxinyu@stu.scu.edu.cn (X.P.); zhouxd@scu.edu.cn (X.Z.)

**Keywords:** drug delivery systems, oral infectious diseases, stimuli-responsive DDS

## Abstract

The oral cavity is a unique complex ecosystem colonized with huge numbers of microorganism species. Oral cavities are closely associated with oral health and sequentially with systemic health. Many factors might cause the shift of composition of oral microbiota, thus leading to the dysbiosis of oral micro-environment and oral infectious diseases. Local therapies and dental hygiene procedures are the main kinds of treatment. Currently, oral drug delivery systems (DDS) have drawn great attention, and are considered as important adjuvant therapy for oral infectious diseases. DDS are devices that could transport and release the therapeutic drugs or bioactive agents to a certain site and a certain rate in vivo. They could significantly increase the therapeutic effect and reduce the side effect compared with traditional medicine. In the review, emerging recent applications of DDS in the treatment for oral infectious diseases have been summarized, including dental caries, periodontitis, peri-implantitis and oral candidiasis. Furthermore, oral stimuli-responsive DDS, also known as “smart” DDS, have been reported recently, which could react to oral environment and provide more accurate drug delivery or release. In this article, oral smart DDS have also been reviewed. The limits have been discussed, and the research potential demonstrates good prospects.

## 1. Introduction

The oral cavity is part of the digestive system, which is composed of many important anatomical structures, including teeth, periodontal tissues, oral mucosa, maxillary and mandibular bones, as well as other soft and hard tissues. It is also a complex ecological niche as more than 700 microorganism species colonize the oral cavity, which is closely associated with oral health [1]. The oral microbiota could help prevent pathogenic microorganisms from growing and help to maintain the stability and balance of oral microecology [2]. However, the composition of oral microbiota could alter due to the change of diet, poor oral hygiene, systemic diseases, etc., which might lead to the dysbiosis of oral microecology [3], and thus many oral microbiota related diseases, i.e., oral infectious diseases. It has been widely considered that oral infectious diseases such as dental caries, periodontitis, peri-implantitis, and oral candidiasis are caused by microbial dysbiosis instead of specific kinds of bacteria [4,5,6]. Furthermore, it has been gradually recognized that oral health is closely related to systemic health.

Therefore, great effort has been made for the treatment and prevention of oral infectious diseases [7,8]. Drug therapy plays an important role in the inhibition of bacterial growth and inflammatory response, and thus the promotion of tissue regeneration [9,10,11]. Systemic administration and local drug delivery are both important ways for drug administration. However, systemic administration could cause many other problems. For example, systemic antibiotics such as tetracycline, beta-lactam antibiotics, nitroimidazoles have been used, especially in cases of periodontal diseases and peri-implantitis [12]. However, systemic antibiotics could cause problems like drug resistance, dysbacteriosis, and systemic side effects [13,14]. The antibacterial effect is also limited as very little could arrive at the oral lesion area after systemic circulation [15,16,17]. Fluoride in drinking water has been used for the prevention of dental caries but might cause excessive intake, leading to fluorosis [18]. Therefore, local drug therapy is now more considered for oral infectious diseases [19,20]. However, the conventional forms of local therapy, like drug suspension or rinse of anti-infection agents, could be easily washed off and thus could not last long in the oral cavity. Complex local lesions like deep periodontal pockets and teeth fissure are also difficult to reach. In order to improve the effect of prophylaxis and treatment, more precise targeting therapy is quite essential [21,22].

Therefore, drug delivery systems have drawn great attention in recent decades in oral infectious diseases. Drug delivery systems (DDS) are devices that can transport and release the therapeutic agents or bioactive substances to certain sites at certain rates in vivo [23,24], usually composed of the carriers and associated therapeutics [25]. They have been widely explored in biomedical research. With local drug administration and controlled drug release, DDS could provide higher curative efficiency and fewer side effects [23,26,27]. With all these advantages, DDS has been reported widely in oral infectious diseases. Numerous kinds of molecular polymers are synthesized as drug carriers [25,28,29]. Bio-adhesive devices like varnish, gels, chips, and tablets were early carriers for the DDS. They could adhere to the mucosa or tooth surface for sustained drug release [30]. Antibiotics or antibacterial agents like chlorhexidine were loaded for local biofilm inhibition [19,31,32]. Remineralizing agents like fluoride were also loaded on varnish or gels for caries prevention and treatment for early caries, which are now commercialized and have been used in clinics [33]. With the application of nanotechnology, micro/nano-scaled carriers are reported in the DDS for oral diseases, which is more comfortable and multifunctional [34]. The carriers could be designed and modified for the loading of anti-bacterial agents [35,36,37], anti-inflammatory drugs [38], and biomolecules like protein and gene, growth factors, which expanded the application of DDS in oral diseases [39,40,41]. These nano-scaled DDS are combined with dental materials like dental restoration systems and dental implants for anti-bacterial modification without affecting their basic properties [42,43]. Further, hydrogel DDS with great biocompatibility and high drug-loading rate have also been applied in oral diseases, especially in periodontal diseases. Injectable hydrogel DDS could be injected into the deep periodontal pocket for sustained topical drug treatment. They could also act as a scaffold for tissue regeneration. Meanwhile, dendrimers poly(amidoamine) (PAMAM), which could promote biomimetic mineralization, have also been used as carriers loading anti-bacterial agents yielding dual-functional DDS with hard tissue regeneration and anti-microbial effect. DDS research has become one of the most important topics in oral biomaterials and provided the implications of new therapeutic strategies for difficult oral diseases [26]. Moreover, in recent years, with the development of the fourth generation of biomaterials, “smart materials” like bio-trigged and stimuli-responsive materials have been introduced to the design of DDS [44,45,46]. The DDS could react to specific conditions of oral cavity such as pH, temperature, enzymes, and provide more accurate drug delivery. These intelligent materials provide more possibilities for oral biomaterials research and the treatment for oral diseases [47,48,49]. 

In this review, we will focus on the recent work and emerging applications of DDS in the prevention and treatment of common oral infectious diseases, including dental caries, periodontitis, peri-implantitis, and oral candidiasis, in order to find out the current challenges and opportunities for future research.

## 2. Carriers of DDS

**Figure 1:**
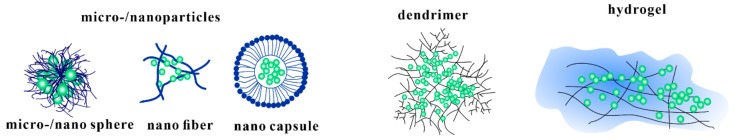
Examples of the structures of different kinds of carriers.

With the rapid development of fabrication of drug delivery carries and loading technology, many types of DDS in oral medicine have been reported. Various kinds of carriers have been applied in different oral diseases according to their character. Table 1 lists various types of DDS carriers reported recently for the treatment of oral infectious diseases. Figure 1 shows some examples of the different kinds of DDS carriers 

### 2.1. Micro/Nanoparticles 

Micro or nanoscale particles, especially nanoparticles, are among the most important carriers of anti-bacterial drugs or other bioactive agents for oral infectious diseases, the dimensions of which are measured in micrometers and nanometers, respectively [27,59]. They are usually manufactured from copolymers such as poly(lactic-co-glycolic acid) (PLGA), poly(D, L-lactide) (PDLLA), poly(ethylene glycol)(PEG) and other biopolymers like lipid, chitosan, pectin, and alginate They are biocompatible, mostly bio-degradable, and could easily be modified or combined for drug loading [44,60]. Many of them have bio-adhesive abilities that could adhere to oral mucosa or tooth surface for sustained local drug release. Some others, like liquid crystalline nanoparticles, could assemble into the gel with improved drug loading and bio-adhesion [39]. They could also be a modified ligand receptor, thus showing the capability of targeting drug delivery [61]. Furthermore, nanoparticles are also used for the modification of dental materials as their nano-scaled structures would not affect the basic properties of materials. They can be fabricated in different forms for different functions. For example, some nanoparticles and nano capsules have been added to dental resin for caries prevention loaded with anti-bacterial agents while nanofibers can be coating on dental implants as since they could act as a scaffold for tissue regeneration. Recently, research has also reported pH-responsive nanoparticles that could respond to the acidic oral environment [42,43]. The nanoparticles modified with pH-sensitive groups such as tertiary amine showed structural changes in acid, thus leading to trigged drug release [47].

### 2.2. Hydrogels

Hydrogels are water-soluble polymers with a highly porous structure. The three-dimensional, cross-linked networks can easily swell in the aqueous environment and imbibe water or biological fluid, thus forming gel matrix [62]. Hydrophilic groups such as –OH, –CONH–, –CONH2– are related to the formation of hydrogel structures [63]. Meanwhile, the porosity structure of hydrogels allows drug loading, and the swelling of hydrogels in aqueous solution leads to controlled drug release. Furthermore, hydrogels demonstrate good biocompatibility as well as similarity with the native extracellular matrix due to the high water content. Most of them had excellent bio-adhesive abilities. Hydrogel DDS could be applied to the tooth surface and oral mucosa as bio-adhesive materials for sustained drug release [64]. In the treatment for periodontitis and peri-implantitis, hydrogel DDS has been widely reported, among which injectable drug delivery systems were newly fabricated hydrogel system [65]. The drug is delivered to the periodontal tissue by a liquid dosage form, and after it reaches the target sites, the form converts into a gel dosage form by the sol-gel transition. And then, the sustained release of the drug can be achieved [66,67,68]. This application can be rapidly carried out without causing pain using a syringe. They can easily be applied in liquid form to the site of drug absorption. Further, at the site of drug absorption, they swell to form a stable gel that is capable of prolonging the residence time of the active substance.

### 2.3. Dendrimers

Dendrimers are three-dimensional globular macromolecules with branches emanating from a central core [69]. Poly(amidoamine) (PAMAM) dendrimers are the first synthesized commercialized dendrimer family. They are fabricated by different methods. With the external reactive groups and controlled spatial structure, PAMAM dendrimers have been used for mimicking natural non-collagenous proteins, which have been widely reported in biomineralization research [70,71]. Meanwhile, with the branch architecture, PAMAM could be loaded with drugs for local drug release [72]. In anti-caries research, anti-bacterial agents like triclosan were loaded on PAMAM. The PAMAM DDS could self-assemble into macroscopic aggregates with the local release of anti-bacterial agents. So, in the prevention and treatment for dental caries, dendrimers have been applied for the fabrication of dual-functional DDS [58].

## 3. DDS for Dental Caries

Dental caries is one of the most prevalent chronic diseases in the world. The 2016 Global Burden of Disease Study demonstrated that caries of permanent teeth ranked No. 1 with the greatest prevalence and No. 2 with the highest incidence [73]. As a common oral infectious disease that could happen at all ages, caries is the major cause of oral pain and tooth loss and represents a severe hazard to human oral health. The WHO has acknowledged that dental caries is a primary health problem in most industrialized countries [74]. 

Dental caries is a dynamic and multifactorial disease occurring on dental hard tissues and is initiated by the acidic by-products from oral biofilms. The etiology and pathophysiology of dental caries are too complex to be entirely clear. It is widely accepted that the local microecology shifts and the break of re-/demineralization of teeth could cause dental caries. Further, saliva, fluoride application, dietary sugars, and preventive behaviors could also affect it [10,75]. In consideration of the harm of dental caries, the timely treatment and prevention are quite essential. It was found that with proper treatment, dental caries can be initially reversible or stopped [76,77]. 

Therefore, efforts have been made for the prevention and control of the disease. Since the dental plaque formation and demineralization of teeth are the major processes of the development of dental caries, anti-bacterial and remineralization agents were widely investigated [78,79]. In recent decades, a combination of drug delivery and controlled release systems with the research and development around caries prevention has gradually drawn attention. They could help to maintain the concentration of anti-caries agents in situ by the sustained drug release. Such local application has many advantages, such as high efficacy and few systemic effects. Thus, there has been an increasing number of researches on DDS for different stages of dental caries in recent years.

### 3.1. DDS for Initial Dental Caries

#### 3.1.1. Antibacterial DDS

**Figure 2:**
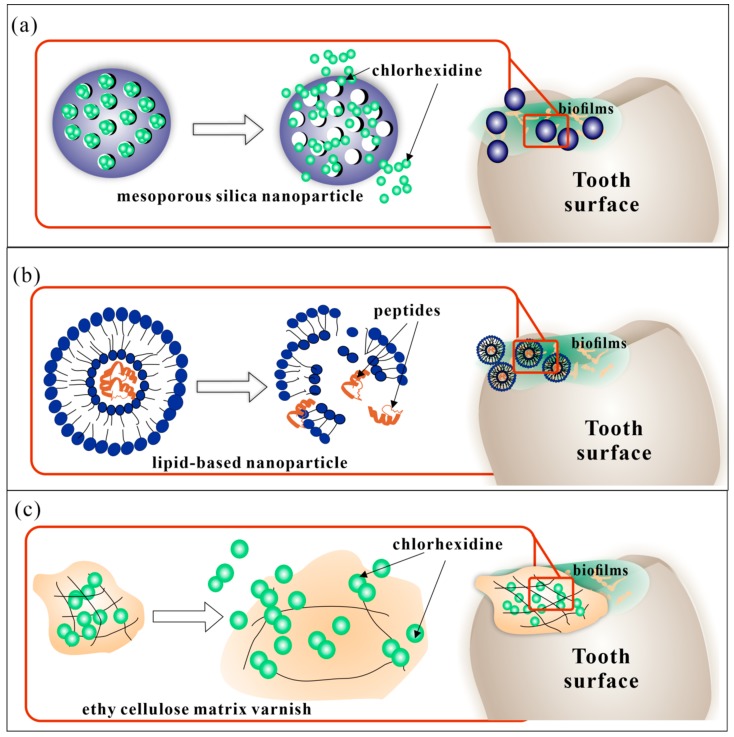
Recent anti-bacterial DDS for dental caries. (**a**) chlorhexidine loaded mesoporous nanoparticles; (**b**) peptides loaded lipid-based nanoparticle; (**c**) chlorhexidine loaded ethy cellulose matrix varnish.

As for the prevention and treatment for early dental caries, noninvasive and drug therapy is the main treatment. Anti-bacterial DDS were investigated widely for the reduction of dental plaque, the initiating factor of dental caries. Different kinds of anti-bacterial agents, such as peptides, chlorhexidine, quaternary ammonium salt, and traditional Chinese medicine were loaded for biofilm removal. BDDS also were applied for local drug delivery in these studies, among which chlorhexidine loaded bio-adhesive systems were most studied [32]. The chlorhexidine loaded varnish was usually ethyl cellulose matrix or other biocompatible copolymers that could sustainably release chlorhexidine (Figure 2c). Some products have already been applied in clinical studies and demonstrated significant anti-bacterial effects that could last from days to weeks. Other BDDS such as chitosan-based propolis varnishes were also investigated. Franca et al. reported the BDDS with sustained release of propolis for a week showed antimicrobial activity similar to or better than chlorhexidine varnish [80]. In recent years the nano-scale DDS have drawn increasing attention. Liposomes, micelles, and other copolymer nanoparticles were used as carriers to conduct the local delivery of anti-bacterial agents such as peptides, triclosan, chlorhexidine [31,35,81] (Figure 2a,b). Several studies have reported lipid-based carriers like liquid crystalline or liposomes loaded with antimicrobial peptide. Such kinds of BDDS could spontaneously form thermodynamically stable lipid bilayers on the bio-interface with biodegradability, excellent biological adhesion, high drug loading, and sustained drug release [82]. For example, Bernegossi et al. reported a decapeptide loaded mucoadhesive liquid crystalline system (LCS). The mixture of PPG-5-CETETH-20, oleic acid, and poloxamer 407 dispersion could aggregate as microemulsions and form liquid crystalline lamellar phase [39]. The KSL-W peptide (F2-P) was incorporated and showed significant anti-biofilm effects against the saliva-derived biofilms. Similarly, peptides such as showed D1–23 and p1025 were also reported recently, with high viscosity and bioadhesion when diluted with artifcial saliva and significant anti-bacterial effect against *Steptcoccus mutans (S. mutans)* biofilms [83,84,85]. Furthermore, gene delivery technology was also applied in the research on DDS for dental caries. Chen et al. designed a novel nanoparticle system loaded with DNA vaccine pGJA-P/VAX, which showed pH-mediated DNA release and enhanced mucoadhesive properties. pGJA-P/VAX could encode a GLU domain of GTF enzymes as well as the A and P regions of a surface protein antigen (PAc) of *S. mutans*, and thus reduce the colonization of *S. mutans*, inducing effective mucosal immune responses [40].

#### 3.1.2. Remineralizing DDS

**Figure 3:**
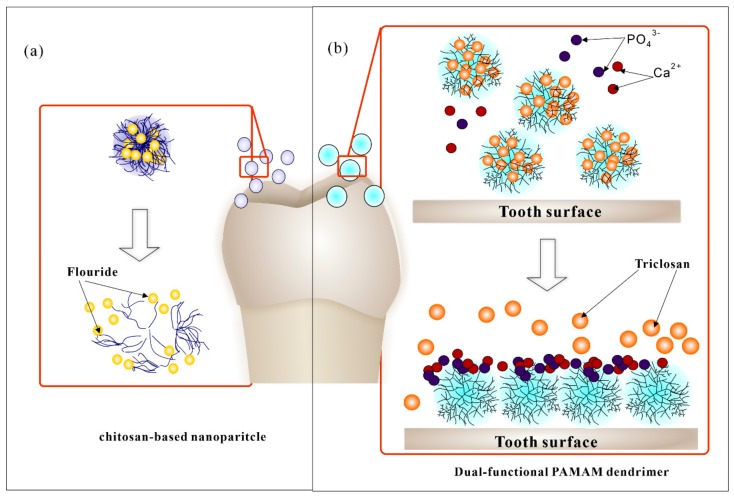
Recent remineralizing DDS for dental caries. (**a**) Flouride loaded chitosan-based nanoparticles; (**b**) Triclosan-loaded PAMAM dendrimer. The dual functional DDS showed anti-bacterial effect and dental remineralization as well. Triclosan is an anti-bacterial agent while PAMAM dendrimers could aggregate into a microribbon structure and promote dental remineralization.

The remineralization agent fluoride has been widely used in the clinic or at home for years, and the fluoride DDS were one of the earliest applications in the prevention of early dental caries. It can form fluorapatite structure on the tooth surface with low acid solubility and thus inhibits demineralization and promote the remineralization [86]. Different kinds of fluoride products have been available for local fluoride delivery, such as toothpaste, mouthwash, varnishes, and gels, etc. [33]. However, such kinds of delivery demonstrate a limited control of sustained release, which could easily be diluted or cleared by saliva and chewing in several hours, so that bioavailability is greatly reduced [87]. In this case, long-term intra-oral released systems were investigated, especially for high-caries-risk individuals [88]. Copolymer or glass devices, bio-adhesives polymers, micro-/nanoparticles, etc. severed as carriers for the delivery and releases. A membrane-controlled reservoir was first reported in 1970, which was a hydrogel copolymer carrier composed of 50/50 hydroxyethyl methacrylate (HEMA)/methyl methacrylate (MMA) copolymer as a fluoride-loaded inner core and a 30/70 HEMA/MMA copolymer membrane. Hydration of the device caused the fluoride release ranging from 0.02 to 1.0 mg per day, which could last to 180 days [54]. Then, other copolymer and glass devices carriers with different concentrations of fluoride and release rates were developed in the following years [89]. Such kinds of DDS were usually attached to the tooth surface by resin adhesives. To improve the comfort and simplify the operation, BDDS were investigated [90,91]. Researchers have reported bio-adhesive tablets with sustained release of fluoride for hours since 1980s [92]. In recent years, novel BDDS produced with nano-technology has been reported (Figure 3a). Keegan et al. synthesized a new kind of bioadhesive chitosan/fluoride microparticles with 6 h sustained fluoride release and acceptable mucosal adhesive ability [93]. Nguyen et al. investigated fluoride loaded nanoparticles based on the biopolymers chitosan, pectin, and alginate. Further, a 4-h steady increase fluoride release was observed. Such kind of nano-BDDS could help improve patient acceptability and compliance, but more sustained fluoride release research and in vivo test is still necessary for the future research [94]. In addition, dual-functional DDS with antibacterial and remineralizing effects have also been reported (Figure 3b). Zhou et al. formulated triclosan-loaded G4-COOH PAMAM [58]. It showed long-term release of anti-bacterial drug and remineralization of dentin, which provides a novel strategy for dental caries and dentine repair at the same time. 

### 3.2. DDS for Secondary Caries

**Figure 4:**
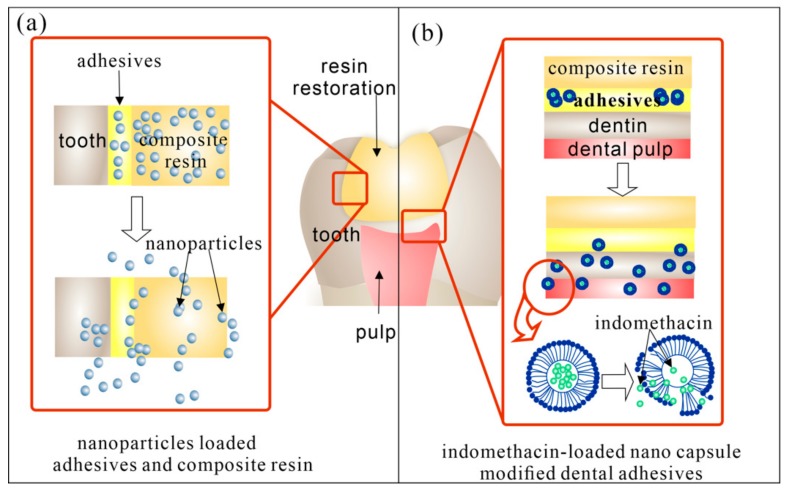
Recent DDS for secondary caries. (**a**) Nanoparticles loaded restoration resin. Adhesives and composite resin could be loaded with anti-bacterial nanoparticle (such as Ag and ZnO) and remineralizating agents like Nano-ACP. (**b**) Indonmethacin-loaded lipid-based nano capsules modified dental adhesives. The nano capsules could release from the adhesives with sustained release of indomethacin which had anti-inflammatory effect on dental pulp.

When dental caries developed to cavitated lesions, remineralization treatment, or another drug therapy cannot help to reverse the situation. So, restoration treatment is necessary. However, dental restoration is faced with many problems such as the high rate of secondary caries and the threat of pulp stimulus. In recent decades, local DDS modified dental restoration demonstrated anti-biofilms, anti-inflammatory, and/or remineralization effect to defend those problems. Figure 4 showed recent research about different kinds of modified dental resin with local drug delivery and release.

Chlorhexidine is still widely used in the DDS modified resin [95,96]. Nanoparticles, sphere, capsules, etc were designed as carriers. For example, Luo et al. synthesized spherical chlorhexidine particles incorporated resin with a controlled release for 650 h [97]. Boaro reported a chlorhexidine/montmorillonite particle, which could release chlorhexidine for 10 days [98]. 

Metal particles are also main antibacterial agents for the local antibacterial modification of dental resin. Silver particles with broad-spectrum antibacterial property are most used [99,100]. Researches have incorporated them into restoration and adhesive resin. But the main drawback of silver particles is the tooth-color effect. So other metal particles such as colorless ZnO was added into the composite. Meanwhile, ZnO has been reported to act as opaque reinforcing fillers in resin [101]. For example, Chen et al. reported ZnO@m-SiO_2_ modified composite resin, which had improved mechanical properties and antibacterial activity [102]. Some researchers have combined silver with ZnO. Previous studies found it more effective than individual components. Other antibacterial agents like triclosan and cationic agents were also loaded [103]. What’s more, to reduce the inflammatory response of pulp caused by deep dental caries, anti-inflammatory agents such as indomethacin have also been combined in the dental resin [104]. Genari et al. reported indomethacin-loaded nano-capsules which could reduce the nociceptive and inflammatory response in vivo [42]. They also combined anti-bacterial agents yielding dual-functional DDS modified dental resin with controlled indomethacin and triclosan release, significant antimicrobial effect without compromising its physicochemical properties [105,106]. The incorporation of remineralization agents is also crucial in the DDS modified dental resin. Calcium phosphate (CaP) composites have demonstrated the controlled release of Ca and P ions for remineralization in an acid environment. The incorporation of calcium phosphate nanocomposites (Nano-ACP) has been reported in dental restoration systems without affecting mechanical properties. Some showed a combination with anti-bacterial DDS, which demonstrated favorable anti-caries effect [107]. 

DDS for prevention and treatment for dental caries have been widely reported. Antibacterial and remineralizing agents have been loaded in different carriers for local sustained and controlled release. Multi-function DDS have then caused great attention. With the development of nanotechnology, modification of dental resin by DDS have also widely studied. For the long-term anti-caries effect of DDS, great efforts have already been made. However, in recent years, increasing emphasis has been put on oral environment balance. Keeping microbial eubiosis instead of killing all biofilms have drawn great attention. Intelligent materials responding to change of oral environment could be a great solution, which will be mentioned in the following. The research is still limited, so there is a great potential for future study.

## 4. DDS for Periodontitis

Periodontitis is a complex infectious disease with several etiologic and contributory factors [108]. It is one of the world’s most prevalent chronic diseases. Researches showed that 743 million people worldwide possess severe periodontitis [109]. The incidence of periodontal disease is closed related to bacterial infection. Bacteria, including its components in dental plaque, is the initial factor and plays a vital role in the process of periodontal disease. According to published reports, *Aggregatibacter actinomycetemcomitans (A. actinomycetemcomitans)* and the red-complex, including *Porphyromonas gingivalis (P. gingivalis)*, *Treponema denticola (T. denticola)*, and *Tannerella (T. forsythia)* are considered closely related to periodontal diseases [110,111]. These bacteria can release bacterial lipid polysaccharides, hydrogen sulfide, ammonia, endotoxin, enzymes (collagenases), and antigens, which lead to periodontal inflammation [22]. This process involves gingival bleeding, periodontal pocket formation, and alveolar bone resorption among other symptoms, leading to the destruction of periodontal tissue [68]. Oral health is also closely related to general health. Therefore, smoking, drinking, stress, diabetes, and so on are all risk factors of periodontitis [112].

At present, the most basic way to treat periodontal disease is to use periodontal scaling and root planning to remove plaque and calculi on the surface of teeth [113]. Because periodontal disease is an inflammatory disease closely related to bacterial infection, to improve the curative effect, periodontal disease management often cooperates with the use of anti-microbial agents and anti-inflammatory drugs. The drugs can be administrated by systemic or local administration [114]. However, owing to its numerous side effects such as antimicrobial resistance, low bioavailability, and systemic adverse reactions, and so on, systemic administration is not an ideal drug administration method [115]. Nowadays, local drug delivery has become a common way of drug administration for periodontal tissue. And the periodontal pocket provides a natural reservoir bathed by gingival crevicular fluid which is easily accessible for the insertion of a delivery device. So, researchers tried to incorporate the drug into different carriers for insertion into periodontal pockets. There have been many studies on local delivery of antibiotics and anti-inflammatory drugs for the treatment of periodontitis. As shown in Table 2, commonly used local drug delivery carriers include fibers, strips, films, injectable gels, microparticles, and nanoparticles. 

### 4.1. Anti-Bacterial DDS for Periodontitis 

**Figure 5:**
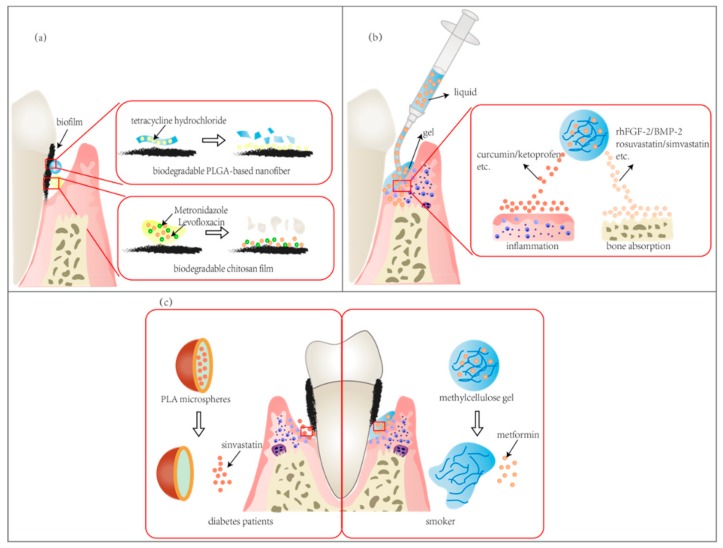
DDS used in periodontitis. (**a**) antimicrobial agents loaded DDS. Various antibiotics (like metronidazole and levofloxacin) can be loaded in drug delivery devices, such as fibers and films. (**b**) DDS for immunomodulation and alveolar bone repair. Injectable drug delivery systems are commonly used devices, and anti-inflammation agents, bone repair factors and osteogenesis drugs are delivered. (**c**) DDS used in periodontitis with risk factors (such as smoking and diabetes).

The mostly used auxiliary method to control periodontal disease is to use antibiotics. Nowadays, various antimicrobial agents have been loaded into different dosage forms to prolong their persistence time in periodontal tissue. Tetracycline group antibiotics, including doxycycline hydrochloride [36,117,125], tetracycline [132,133], and minocycline [122,124]; nitroimidazoles antibiotics like metronidazole [116,120] and tinidazole [55] are mostly used. Other antibiotics like quinolones have also been reported (Figure 5a).

Moura et al. reported doxycycline loaded PLGA microspheres with the sustained release of drugs in the periodontal pocket for 20 days by clinical research [36]. Phaechamud et al. developed doxycycline hyclate-loaded in situ forming microparticles, which exhibited a sustainable drug release for 47 days with Fickian diffusion and effectively inhibited *P. gingivalis*, *S. mutans* and *Staphylococcus aureus (S. aureus)* [129]. DDS for periodontal rinse has demonstrated a great anti-biofilm effect. However, for better treatment and tissue repair, scaffold carriers have been applied. An osteoconductive drug delivery system composed of apatitic nanocarriers was described. It was capable of providing sustained delivery of tetracycline, and the nanocarrier itself can promote bone regeneration [132]. Other storage-type drug delivery systems like fibers, films, and strips were also placed in the periodontal pocket for local sustained release [19]. Marziyeh et al. designed drug delivery devices which were fabricated via blend electrospinning and coaxial electrospinning using PLGA, gum tragacanth (GT), and tetracycline hydrochloride (TCH) as a hydrophilic model drug. Drug release was sustained for 75 days with only 19% of burst release within the first 2 h [133]. Ofloxacin loaded poly (methacrylic acid) and hydroxypropyl-cellulose strips were reported by Kimura et al. [134]. Gayasuddin et al. designed an intra-pocket, biodegradable film of chitosan loaded with metronidazole and levofloxacin meant for inserting into periodontal pockets to treat infections, and it demonstrated sustained release for up to seven days [120]. These studies show that the application of DDS in periodontal pockets had significant therapeutic effects for periodontitis. To prolong action time and improve the operating property, injectable hydrogel DDS were also applied. Yu et al. designed a tinidazole-loaded mPEG–PDLLA hydrogel. This in situ gel forming system showed sustained tinidazole release over 192 h with a low burst effect (around 7% in the first 8 h) in the in vitro release study [55]. Kilicarslan et al. reported in situ forming implants for the local delivery of metronidazole to periodontal pockets. It consisted of capped PLGA and *N*-methyl-2-pyrolidone as solvents with sustained drug released over 10 days [128]. Also, there are some products commercially available, such as Periochip insert (chlorhexidine gluconate, crosslinked hydrolyzed gelatin) and the injectable formulations Attridox (doxycycline, poly-DL-lactide), Arestin(minocycline, polyglycolide-co-DL-lactide), and Elyzole (metronidazole benzoate in glyceryl mono-oleate/sesame oil) [22,135,136,137]. 

### 4.2. Inflammation Modulating and Alveolar Bone Repairing DDS for Periodontitis

Periodontitis is a process of the inflammatory response and can lead to the absorption of alveolar bone. So, to control periodontitis, there are also drug delivery systems for immunomodulation and alveolar bone repair, which usually load drugs that have anti-inflammatory effects or promote bone repair (Figure 5b). Moreover, the most used devices are films, while the second are injectable drug delivery systems.

Curcumin is a commonly used anti-inflammation drug in periodontitis for its anti-inflammatory, antioxidant, antimicrobial, immunostimulant, antiseptic, and antimutagenic properties. Curcumin often acts as an anti-inflammatory agent [138]. Chauhan et al. prepared a curcumin loaded biodegradable crosslinked gelatin film. The optimized film could provide proper mucoadhesive strength, mechanical properties, folding endurance, and could efficient delivery in a sustained manner up to 7 days [119]. Nasra et al. developed in situ gel formulations of curcumin using 30% of pluronic F127, and 1% of carbopol P934. The selected formulations delivered into the periodontal pocket were verified in clinical research [123]. Nanoparticles [38] and gels [139] DDS were also developed for local anti-inflammatory therapy, too.

Other anti-inflammation agents were used to treat periodontitis, such as non-steroid anti-inflammatory drug—ketoprofen, a congener of the 2-arylpropionic acid class of non-steroidal anti-inflammatory drugs (NSAIDs). A therapeutic value was reported in the treatment of periodontal disease via the inhibition of the cyclooxygenase enzyme, which is closely related to the biosynthesis of prostaglandins and inflammatory response of the body. Scrivastava et al. formulated an in-situ gelling injectable nano-emulgel with the sustained release of ketoprofen into the periodontal pocket [127]. Antibiotics and anti-inflammatory drugs can also be co-loaded into a drug delivery system. For example, Sundararaj SC et al. developed a multiple drug delivery system, which can load four types of drugs and achieve the sequential release of drugs. The four types of drugs used were metronidazole, ketoprofen, doxycycline, and simvastatin to eliminate infection, inhibit inflammation, prevent tissue destruction, and aid bone regeneration, respectively [140]. Fisher PD. et al. developed an in situ forming implants to deliver doxycycline and simvastatin [141]. 

Alveolar bone resorption is one of the symptoms of periodontitis. Therefore, to treat periodontitis, drugs which can promote bone repair are used. Therapeutic drugs, such as bone repair factors and osteogenesis drugs, are clinically common.

Some scholars have used drug delivery systems to load bone repair factors for the promotion of bone regeneration in periodontal tissues. To evaluate if a biological hydrogel of recombinant human fibroblast growth factor type 2 (rhFGF-2) in a hyaluronic acid (HA) carrier can play a role in regeneration of the periodontal attachment apparatus, a randomized controlled clinical trial was carried out. Hereby, it was concluded that the application of rhFGF-2/HA significantly improved the clinical parameters of periodontal wound healing one year after treatment [56]. Also, it was combined with bone cement by other researchers. Oortgiesen et al. designed an injectable macroporous calcium phosphate cement (CaP) in combination with bone morphogenetic protein-2 (BMP-2) or fibroblast growth factor-2 (FGF-2) [41]. They also combined enamel matrix derivative with an injectable calcium phosphate cement [142]. Both approaches can significantly promote periodontal regeneration.

Osteogenesis drugs were also loaded in the DDS for bone defection. Statins such as rosuvastatin and simvastatin stimulate in vivo bone formation, increasing new bone volume. Pradeep et al. reported 1.2% rosuvastatin (RSV) gel incorporated into a methylcellulose vehicle to intrabody defect sites, and it showed a greater reduction in probing depth and gingival index, along with increased gain in clinical attachment level [143]. The effect of 1.2% simvastatin gel as a local drug delivery system on Gingival Crevicular Fluid (GCF) Interleukin-6 (IL-6) and Interleukin-8 (IL-8) levels was evaluated. In adjunct to SRP, 1.2% Simvastatin gel could be an effective agent for the management of chronic periodontitis [144].

The progression of periodontitis involves a complicated, sequential relationship between infection, inflammation, and tissue loss, and thus combining different drugs in drug delivery systems can achieve comprehensive treatment. Therefore, further research on muti-functional DDS would have great potential for clinic application.

### 4.3. Treatments of Periodontitis Associated with Systemic Diseases

Oral diseases are closely related to general health. Smoking and diabetes are both risk factors for periodontal disease. Recearches showed that DDS can be also used to treat periodontitis with these risk factors (Figure 5c). 

Studies have shown that smokers generally have a high risk of periodontal disease [145]. So, the treatment of periodontitis among smokers has caught great attention. It was demonstrated that drug delivery systems were effective in the treatment of smoker’s periodontitis. Rao et al. developed an indigenously prepared, biodegradable, controlled-release gel which was loaded with metformin. It was used in the treatment of vertical defects in smokers with generalized chronic periodontitis. Moreover, the results showed that the gel formula had a better clinical therapeutic effect [146]. Also, simvastatin was loaded in the DDS which showed significant promotion of bone fill in smokers with periodontitis [147]. Chandra et al. used lycopene as a locally delivered gel to treat periodontitis and compared its efficiency in smokers and nonsmokers, and there was no statistically significant difference in the clinical parameters [148]. 

Diabetes is another critical systemic disease closely related to periodontitis [112]. It can promote the development of periodontal disease and aggravate the severity of periodontal infections [149]. So, there are many DDS used for the treatment of patients with chronic periodontitis with diabetes mellitus. Animal experiments and clinical trials were carried out to evaluate their efficiency. Pradeep et al. designed double-masked clinical trials to investigate the effectiveness of locally drug delivery systems, like 1.2% simvastatin in situ gel (methylcellulose) [150] and 1% alendronate gel (carbopol 934P and triethanolamine) [151]. Both formulations showed a significant increase in PD reduction, CAL gain, and improved bone fill. They could be used as an adjunct to SRP to provide a new dimension in the periodontal therapy soon. Also, Li et al. prepared 25-hydroxyvitamin D3-loaded polylactic acid (PLA) microspheres and established a diabetic periodontitis model. They used the microspheres to treat the rat, founding that alveolar bone loss was inhibited, and osteoid formation in the periodontium was promoted [152]. All the researches showed us that DDS could provide an effective approach for the treatment of diabetic periodontitis. 

## 5. DDS for Peri-implantitis

Since 1970s, when the first titanium root-form implant was reported, dental implantation has been developing rapidly and become one of the most important therapy for detention defects [153]. With the increasing number of dental implants applied in clinic, evidence has been revealed that peri-implant infection and inflammation could significantly affect the surrounding soft and hard tissues leading to the failure of the implant [154]. Peri-implantitis is defined as the inflammatory process of the implant-surrounding hard and soft tissues, accompanied with bone loss, decreased osseointegration and periodontal pocket [155]. It is reported that about 50% of mucositis appears in dental implants, while 28-56% of patients suffer from peri-implantits [156]. Peri-implant diseases are multifactorial diseases. Smoking, systemic diseases such as diabetes and immunosuppression, history of periodontitis, history peri-implants disease, and etc. could be risk factors [154]. However, microbial colonization is considered as the main factor in the etiology. Like many other infectious oral diseases, peri-implantitis are also considered as poly-microbial infection. Some periodontitis related microbiota have been reported closely related to peri-implants diseases such as *Prevotella intermedia (P. intermedia), A. actinomycetemcomitans*, *P. gingivalis*, *T. denticola*, and *T. forsythia*. What’s more, *S. aureus, Candida albicans (C. albicans)* have also been detected [156,157,158]. 

The local treatment of peri-implantitis composed of non-surgical and surgical therapy is most commonly used to control infections and/or improve osseointegration [159,160]. With the sustained drug release and better biocompatibility, DDS could be suitable for the treatment of peri-implantitis. Since there are some similarities in etiology and risk factors between peri-implantitis and periodontitis, some DDS for periodontal treatment could also be referred to the peri-implantitis. Antibiotics or antibacterial agents, such as tetracycline, minocycline, chlorhexidine, were loaded to carriers like chips, gel, microsphere, polymeric fiber [161,162,163]. Some products like Arestin^®^ [164], perioChip^®^ [165], and Atridox^®^ [166] for periodontitis also showed great application potential in peri-implantitis. Recently, dual-function DDS with antibacterial and bone regeneration effect has been reported as well. Diniz and co-researchers have a silver lactate loaded hydrogel, which could help induce Osteogenic differentiation of GMSCs and hBMMSCs, and significantly inhibit *A. actinomycetemcomitans* with releasing silver ions for up to 2 weeks [167].

Although great efforts have been made for peri-implantitis, which have brought significant effect, there are still problems, such as osseo-re-integration. Therefore, prevention of peri-implantitis and improving osseointegration after implantation have drawn great attention, especially when the surface treatment technology of the implants was greatly improved in recent years [168]. Anti-bacterial materials, antibiotics, and other bioactive agents loaded DDS were coated to implant surface by topographical modifications, biodegrading polymer incorporation, and so on [153,168,169]. Such kinds of special DDS for dental implant have been fabricated with significant anti-bacterial or bone regeneration effect for the sustained drug release. Table 3 listed the recent researches on anti-bacterial DDS coating for the prevention of dental implant diseases. Anti-bacterial peptide, chlorhexidine, antibiotics like doxycycline and tetracycline were incorporated into dental implants by covalent conjugation. Nanoparticles, widely used in DDS fabrication, were also applied in coating dental implants. Metal irons such as zinc oxide and silver nanoparticles are most used in the coating [50,51]. They were incorporated into the titanium and abutments by electrodeposition technology. One-species [170], multi-species [171], saliva biofilms [172], and in vivo [173] test were applied for the antibacterial effect and biocompatibility. Other bioactive nanomaterials can also be coated with dental implants as carriers such as biodegradable and/or bioactive agents gelatin, chitosan, and hydroxylapatite, which can also help the bone regeneration. A. Besinis et al. reported a dual layered nanocoating comprising silver, titanium dioxide, and hydroxyapatite [174]. Bottino et al. fabricated a gelatin nanofiber loaded with tetracycline, which demonstrated great anti-bacterial effect and induced the growth of osteo-precursor cells [43]. Furthermore, researchers found out paramagnetic implants can promote the osteogenic response of pre-osteoblast cells. Yang et al. reported a PLGA(Ag-Fe3O4)-coated dental implant with antibacterial effect and promotion of osteoblast proliferation as well [175]. 

Such kinds of dual-functional DDS not only show antibacterial effects without affecting biocompatibility, but also help promote bone regeneration, which has excellent application potential. However, long-term drug release and multifunctional dental implant research are still limited. Further researches were still necessary for the future.

## 6. DDS for Oral Candidiasis

Oral candidiasis is a common fungal disease of the oral cavity mainly caused by candida infection [185]. *C. albicans* is the most common species isolated in the oral candidiasis. It is an opportunistic infection, as candida is present among normal oral microbial flora, which could be detected in 30% to 55% of healthy adults. Local and systemic problems could cause an overgrowth of Candida species and oral candidiasis, such as defective prosthesis, misuse of denture, long-term use of corticosteroid and broad-spectrum antibiotics, immunodeficiency, and etc. [186].

**Figure 6:**
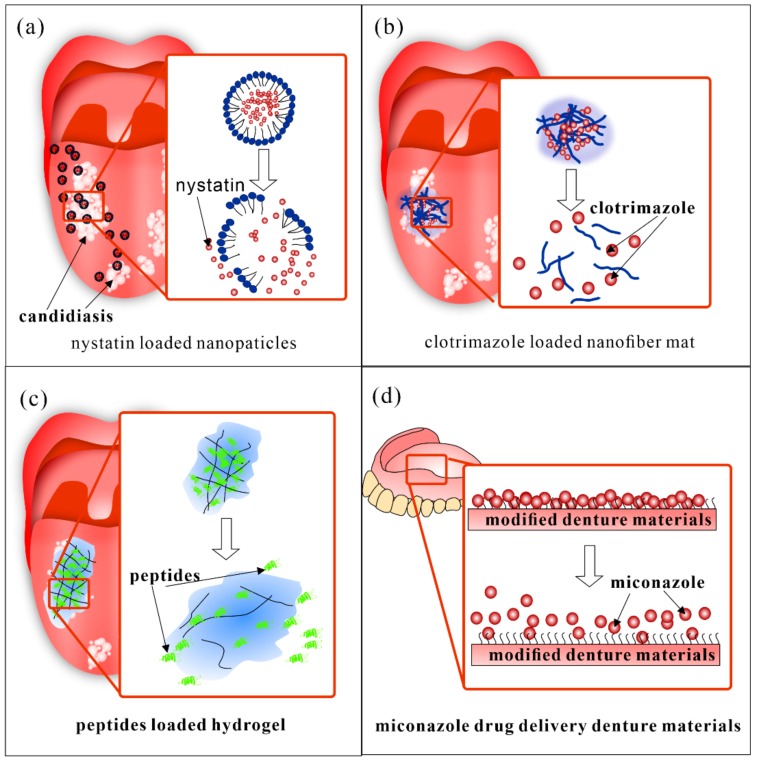
Recent DDS for oral candidiasis. (**a**) nystatin loaded lipid based nanoparticles, (**b**) clotrimazole loaded nanofiber mat; (**c**) Peptides loaded bioadhesive hydrogel; (**d**) modified denture materials with sustained drug release. The copolymer modified denture could had improved binding with miconazole and thus showed sustained drug release.

Taking into account the high toxicity and the threat of drug resistance of systemic treatment, local drug delivery has been used widely. Oral suspensions like clotrimazole troches (CT) and nystatin suspension have been recommended as a first-line treatment of uncomplicated oral candidiasis by the Infectious Diseases Society of America, but there are still some problems remained to be solved, such as short contact time with the oral mucosa and frequent daily dose [187]. Thus, DDS have been researched in recent years. Different kinds of bio-adhesive devices have been applied, such as clotrimazole lozenges, nystatin tablets, miconazole buccal tablets, and so on [188,189]. A miconazole mucoadhesive tablet has been reported, which had in vitro antifungal activity against many Candida species. With a once-daily application of the tablet containing 50 mg of miconazole of 50 mg, it could maintain high levels of salivary miconazole concentrations effectively treat pseudomembranous candidiasis [190]. Other nano-scaled copolymers, lipid, or chitosan-based DDS have also been investigated (Figure 6a–c). 

Fernandez et al. reported a nystatin-loaded nano-emulsion for the buccal treatment of candidiasis, which could slowly release of nystatin and high efficiency in vitro antifungal effect with no systemic nystatin concentration or damage in mucosa ultrastructure [37]. Kenechukwu et al. investigated a miconazole nitrate-loaded nano lipid gel which possessed desirable stability, readability as well as better prolonged release and anti-fungal properties [191]. Zhou et al. prepared AmB/MPEG-PCL-g-PEI (amphotericin B/monomethoxypoly (ethylene glycol)- poly (epsilon-caprolactone) -graft-polyethylenimine, MPP) micelles loaded tablet with 8-h slow release, reduced cytotoxicity, and improved anti-biofilm effect in vitro [192].

Tonglairoum et al. fabricated a clotrimazole loaded nanofiber mat coated with chitosan, which presented good mucoadhesive properties and killed Candida more efficiently than the commercial CZ lozenges at 5, 15 and 30 min in vitro [53]. A similar kind of Miconazole nitrate loaded nanofiber film was also reported by Tejada et al. [193]. Furthermore, Rençber et al. developed chitosan-nanoparticles containing fluconazole for the local treatment of oral candidiasis. The in vivo and in vitro test showed this DDS had the antifungal efficacy and successfully treated the infection with local administration once a day [52].

Some natural compounds and alternative therapies have also been applied to antifungal treatment. Curcumin-loaded polymeric nanoparticles were demonstrated to improve the antimicrobial effect of photodynamic therapy for oral candidiasis [194]. Antimicrobial peptides loaded BDDS were also reported and presented a controlled release and antifungal activity in vitro and in vivo [57]. 

Oral candidiasis that associated with complete or removable partial dentures in the elderly called denture stomatitis is an important part of the research (Figure 6d). Some researchers have worked on the modification of denture for pre-venture and treatment. Czerninski et al. reported a sustained release clotrimazole varnish. In the clinic study, the varnish was applied to the denture once a day and the candida level was significantly reduced after 14 days of treatment [195]. Modified drug delivery denture was also a novel treatment for oral candidiasis [57]. Wen et al. showed denture surface grafted with miconazole loaded polymers with sustained drug release and robust biofilm controlling effect [196]. Further studies on the evaluation of relevant properties are needed.

## 7. The Newly Developed Stimuli-Responsive DDS

### 7.1. The Characteristic of Oral Environment

The oral cavity is a unique ecosystem with multiple anatomic microniches presenting complex physicochemical features, such as pH, oxygen, temperature, or redox potential [197]. In the physiological environment, saliva has a normal pH range of 6.2–7.6, with 6.7 being the average pH [198], and the temperature is around 37 °C. Meanwhile, as mentioned before, oral cavity is colonized by huge amounts of microorganisms. In the healthy state, these characteristics are in a stable state. However, when infectious diseases happen, stability is disturbed, and physicochemical features will be changed. The composition of the microbial community would also shift [4]. The average temperature of the periodontal pocket has been reported to be 36.6 °C ± 0.4 °C [199]. The pH of the infectious disease sites usually drops significantly [200]. At the sites of active caries, the pH can be about 4.5–5.5 with the dominance of acid-producing and acid-resistant bacteria [201]. The periodontitis and inflammatory response caused by subgingival plaque could lead to a drop of pH to around 6.5 [198]. It has also been reported that the pH level around the peri-implant infection surface decreases as low as pH 5.5 [202].

### 7.2. The Stimuli-Responsive DDS for Oral Infectious Diseases

**Figure 7:**
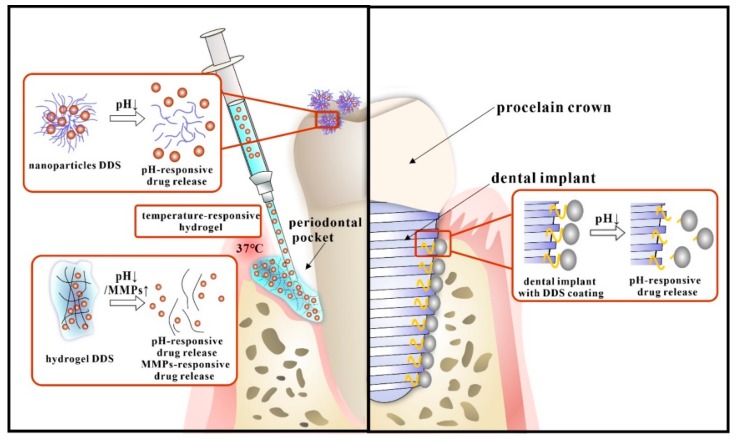
Recent stimuli-responsive DDS in oral infectious diseases.

With the unprecedented progress of biomedical technology, a myriad of novel stimuli-responsive materials has been developed to serve as drug carriers. These kinds of DDS could respond to the shift of environment, including pH, pressure, temperature, oxygen, etc., and thus control the release behavior of payloads, making the drugs “smart” [203]. As for the unique characteristics of the oral microenvironment mentioned before, newly developed stimuli-responsive DDS have excellent prospects for specific therapeutic effect. Although stimuli-responsive DDS have been widely studied in biomedicine. Research on oral stimuli-responsive DDS is limited. Recently, isolated studies (Figure 7) have been reported based on the specific physiological and anatomical characteristics of oral infectious disease. 

There are mainly two kinds of stimuli-responsive drug delivery systems in oral medicine. One is the nano DDS, among which pH-responsive DDS are most used in the oral cavity. The DDS carriers were modified with pH-sensitive groups like tertiary amines or acid-labile bonds. The drug release was manipulated by protonation/deprotonation reaction or rupture chemical bonds during the change of pH [204]. 

The unique acid environment of dental caries is quite suitable for pH-responsive nanoparticles. Horev et al. designed farnesol loaded nanoparticles composed of cationic poly(dimethylaminoethyl methacrylate) (p(DMAEMA)) coronas and hydrophobic and pH-responsive p(DMAEMA-co-BMA-co-PAA) cores, and in the acidic environment caused by bacterial infection, farnesol was released rapidly due to protonation of DMAEMA and PAA residues within nanoparticle cores and resulting destabilization of nanoparticle structure. Further, 75% of the drugs can be released at pH 4.5 for 12 h [47]. Then Zhou and co-workers optimized the nanoparticles by alterations in core molecular weight ratios (CCR) based on previous studies. The results showed that farnesol loaded nanoparticles with higher CCR, had enhanced pH-responsive drug release and thus exhibited greater antibiofilm efficacy in situ [158]. Behrens also reported chlorhexidine loaded nanoporous silica nanoparticles with the pH-activated release [205]. Furthermore, researchers found out that pH-responsive nanoparticles could also be used to treat peri-implantitis, Dong et al. designed a pH-dependent AgNPs releasing titania nanotube arrays (TNT) implant for peri-implant infection control. AgNPs were grafted on TNT implant surface via a low pH-sensitive acetal linker. The acetal linker was stable at neutral pH and could be broken at pH 5.5 so that AgNPs were released from TNT-AL-AgNPs implant in high dose [206].

Furthermore, the shift of infection related factors can also be used as the stimuli for the DDS. Matrix metalloproteinases (MMPs) are a group of enzymes capable of degrading almost all ECM proteins. They also play a marked role in oral infectious diseases, such as periodontitis, peri-implantitis, dental caries. The expression and activity of MMPs in healthy adult tissues is quite low, but in those inflammatory diseases, they can be activated and upregulated [207]. Therefore, MMPs could act as stimuli for novel DDS. Guo et al. designed an MMP-8-responsive hydrogel and demonstrated that it was a promising candidate for on-demand intraoral localized drug delivery [48]. 

The other important kind of stimuli-responsive DDS is injectable DDS, mostly hydrogels, based on the Sol-Gel method. They could also be motivated by the change of environment like pH and temperature, as previously described. They were mostly used in the treatment of periodontal diseases. Chang et al. developed a thermo-gelling and pH-responsive drug carrier that can respond to temperature and pH changes, which was beneficial for treating periodontitis. The hydrogel was made of amphipathic carboxymethyl-hexanoyl chitosan (CHC), β-glycerol phosphate (β-GP), and glycerol. It was consistently fluidic at 4 °C, but rapidly gelled at 37 °C. At the same, due to the protonation of amine groups in an acidic environment, the release of naringin was faster at pH 5.5 [49]. Wang and co-workers studied a novel thermos-responsive polyisocyanopeptide (PIC)-based hydrogel as an injectable carrier for local drug delivery for periodontal applications. They are liquid at low temperatures and thus can be easily injected into deep pockets, and then, they can form gels under body temperature to facilitate sustained drug release [208]. It also can be used in other infective diseases. Harish et al. a developed pH triggered in situ gel for the local release of clotrimazole, using carbopol and ion triggered gellan gum. This could be used in the buccal cavity for the treatment of oropharyngeal candidiasis. In the slightly acidic conditions (pH 6.8) of the buccal cavity, the formulations could release Ca2+ to ensure reproducible gelation of the gellan gum [209]. 

Nowadays, there have been many smart drug delivery systems designed by different principles in the oral cavity. However, researches on these systems are still limited in number and are restricted to fundamental studies, and their clinical application still faces many challenges, such as sophisticated design and synthesis, challenging to maintain a long-lasting effect. Also, further in vivo studies and animal experiments are needed to prove its good biocompatibility. Nevertheless, the application of smart drug delivery systems cannot be neglected to improve the effectiveness of drugs on oral diseases. 

## 8. Prospective in the Future

In the past decades, DDS have developed so quickly that application of DDS it has become an important therapy in medical treatment, with enhanced effect and lower side effect. In the treatment for oral infectious diseases, researches have explored quite many applications to inhibit bacterial growth and biofilm formation, reduce inflammatory response or tissue regeneration. Especially, antibacterial agents loaded DDS has been widely studied and showed sustained bacteriostasis. Remineralization agents and bone regeneration factors or agents have also been loaded for hard tissue repairing. Some other DDS have been incorporated into dental materials for therapeutic modification. These studies show that DDS have great potential for application in the treatment and prevention of oral infectious diseases. Furthermore, some researchers have already reported dual-functional DDS for better efficacy and less dosage. 

Although DDS research has drawn great in oral medicine, there are still many limitations and blanks in this field. For further clinic application, more in vivo researches and long-term studies are also need. In the future, multifunctional drug delivery, which could combine diagnoses with treatment for oral infectious diseases, may have a great prospect. Moreover, since the oral cavity is a unique niche in the human body with a complex micro-ecology, to specifically respond to the change of the oral environment, stimuli-responsive DDS were fabricated. Such intelligent systems could be an important solution for the prevention of drug resistance and oral dysbiosis, which might happen during the anti-infection treatment. However, studies remain limited. How to realize the drug reloading and reversibility for long-term effect for oral infectious diseases is still an unsolved problem. Further researches are desired in the future. It is believed that with the development of materials technology and research on oral infectious diseases, DDS will be optimized and widely applied in a clinical context.

## Figures and Tables

**Table 1:** Carriers of drug delivery systems(DDS) and the application in oral infectious diseases.

Types of Carriers		Application Oral Diseases	Composition
micro/nano particles	micro/nano sphere	dental caries [31,35] periodontitis [38] implant coating [50,51] oral candidiasis [52]	PLGA, PDLLA, PEG and biopolymers like lipid, chitosan, pectin, and alginate
	nanofiber	periodontitis [19] oral candidiasis [53] implant coating [43]	
	nano capsule	dental resin modification [42]	
hydrogel		dental caries [54] periodontitis [55,56] oral candidiasis [57]	Hydrophilic groups such as –OH, –CONH–, –CONH2–
dendrimer		dental caries [58]	PAMAM, poly(aryl ether)

**Table 2:** Local DDS in periodontitis.

Devices	Polymers *	Drug	Suistained Release Time
fibers	PLGA ^1^,GT ^2^ [36]	tetracycline hydrochloride	75 days
	biodegradable polydioxanone [116]	metronidazole or ciprofloxacin	over 7 days
rings/ strips	trimethylene TMC ^3^/CL ^4^,GL ^5^/CL [117]	doxycycline hyclate	28 days
films	PEGylated rosin derivatives(PRDs) [118]	sparfloxacin	over 21 days
	Gelatin [119]	curcumin	up to 7 days
	Chitosan [120]	Metronidazole, levofloxacin	up to 7 days
	PEO ^6^, PDLLA ^7^ [121]	Lipoxin A4	48h
	chitosan, PVA [120]	doxycyclin	over 1 week
in situ gel/ impants	PLGA, NMP ^8^ [122]	Minocycline	over 48 h
	Pluronic F127, carbopol P934 [123]	curcumin	over 1 week
	Pluronic, Carbopol [124]	meloxicam or minocycline HCl	3 days for MH, 7 days for Mx
	Cholesterol, NMP [125]	doxycycline hyclate	10 days
	NMP, Ethylcellulose, bleached shellac, Eudragit RS [126]	NMP	/
	mPEG-PDLLA [55]	tinidazole	192 h
	Carbopol 934P, Polaxamer 407 [127]	ketoprofen	over 24 h
	PLGA, calcium phosphate Cements [41]	BMP-2, FGF-2	/
	PLGA, NMP [128]	metronidazole	over 10 days
micro-/ nano-particles	BS ^9^, NMP, DMSO ^10^, GMS ^11^ [129]	doxycycline hyclate	47 days
	PLGA [36]	doxycycline hyclate	at least 15days
	Chitosan [130]	clindamycin phosphate	more than 1 week
	PEG ^12^, PLA ^13^, RGD peptide [131]	minocycline	14 days
	Nanoparticles [132]	tetracycline	over 5 days

* The polymer carriers of DDS were showed in abbreviation: ^1^ PLGA: poly lactic glycolic acid; ^2^ GT: gum tragacanth; ^3^ TMC: trimethylene carbonate; ^4^ CL: caprolactone; ^5^ GL: glycolide; ^6^ PEO: poly(ethylene oxide; ^7^ PDLLA: poly(d,l-lactide); ^8^ NMP: N-methylpyrrolidone; ^9^ BS: Bleached shellac; ^10^ DMSO: dimethyl sulfoxide; ^11^ GMS: 2-pyrrolidone, glyceryl monostearate; ^12^ PEG: poly(ethylene glycol); ^13^ PLA: poly(lactic acid).

**Table 3:** Anti-bacterial DDS coating for the prevention of dental implantitis.

Coating Type		Anti-bacterial Experiment Model *	Results
Peptide	GL13K-peptide coated titanium [176]	*Pg*^1^; *Sg*^2^	inhibited biofilm growth
	antimicrobial peptide OP-145 coated titanium [177]	*Sa*; in vivo rat model	showed antimicrobial effect, sustained release for 30 days; prevented implant infections
Metal particles	AgNP-doped silica coated titanium [50]	*Aa* ^3^	showed antibacterial effect for at least 7 days
	zinc oxide and hydroxyapatite coated titanium [51]	Human saliva biofilm model	showed antimicrobial effect; reduced facultatively anaerobic and *Streptococcus* spp.
	metal nanoparticle incorporated glassy zirconia abutments [173]	In vivo dog model	prevented biofilm formation and the peri-implant bone loss
	a combination of silver, titanium dioxide and hydroxyapatite nanocoatings [174]	*Ss* ^4^	showed antimicrobial effect; healing.
	Ag-doped calcium deficient dydroxyapatite coated titanium [178]	*E coli*^5^, *Sa*^6^	showed antibacterial effect and sustained release over 14 days
	PLGA(Ag-Fe3O4)-coated dental implants [175]	*Sm* ^7^	inhibited bacteria adherence
	Ag nanoparticles coated on titanium [179]	*E coli*, *Sa*	showed antibacterial effect and sustained release for 7 days
			
Antibiotics	doxycycline-coated abutment surfaces [180]	*Se* ^8^	inhibited the bacterial growth; showed sustained release for least 2 weeks
	Tetracycline-containing fibers coated titanium implant [171]	*Pg*, *Fn*^9^,*Pi*^10^, *Aa*	showed inhibition of biofilm and kept releasing for 3 days
	silica-gentamycin coated titanium implant [170]	*Sa*	showed antibacterial effect and sustained release for 14 days
	Tetracycline loaded nanofibers coated titanium implant [43]	*Aa*, *Fn, Pg*, *Pi*	Showed anti-bacterial effect
	Tetracycline loaded titanium [181]	*Pg*	showed antibacterial efficiency and sustained release for 15 days
			
Cationic antibacterial agents	chlorhexidine hexametaphosphate nanoparticles coated titanium [182]	*Sg*	demonstrated antibacterial effect and sustained release of soluble chlorhexidine for 99 days
	The PIXIT implant containing polysiloxane oligomers and chlorhexidine gluconate [183]	Clinic trail	controlled bacterial adhesion; reduced the bacterial species involved with long-term failure of dental implant
	Dimethylaminododecyl Methacrylate(DMADDM) coated dental implant [172]	saliva-derived biofilm	inhibited biofilm growth and regulated microecosystem
Bioactive antibacterial agents	Chitosan/P-HAP bi-layers coated titanium implant [184]	*Sg*	Demonstrated an appropriate mouse pre-osteoblastic cell response, and significant anti-bacterial activity

* The bacterial model were showed in abbreviated form: ^1^*Pg*, *Porphyromonas gingivalis*; ^2^*Sg*, *Streptococcus gordonii*; ^3^*Aa*, *Aggregatibacter actinomycetemcomitan*; ^4^ *Ss*, *Streptococcus sanguinis*; ^5^ *E coli*, *Escherichia coli*; ^6^ *Sa*, *Staphylococcus aureus*; ^7^ *Sm*, *Streptococcus mutans*; ^8^ *Se*, *Staphylococcus epidermidis*; ^9^ *Fn*, *Fusobacterium nucleatum*; ^10^ *Pi*, *Prevotella intermedia*.
